# Using Photovoice as a Community Based Participatory Research Tool for Changing Water, Sanitation, and Hygiene Behaviours in Usoma, Kenya

**DOI:** 10.1155/2015/903025

**Published:** 2015-08-25

**Authors:** Elijah Bisung, Susan J. Elliott, Bernard Abudho, Diana M. Karanja, Corinne J. Schuster-Wallace

**Affiliations:** ^1^Department of Geography and Environmental Management, University of Waterloo, 200 University Avenue West, Waterloo, ON, Canada N2L 3G1; ^2^United Nations University Institute for Water, Environment and Health (UN-INWEH), Hamilton, ON, Canada L8P 0A1; ^3^Center for Global Health Research, Kenya Medical Research Institute (KEMRI), Kisumu, Kenya

## Abstract

Recent years have witnessed an increase in the use of community based participatory research (CBPR) tools for understanding environment and health issues and facilitating social action. This paper explores the application and utility of photovoice for understanding water, sanitation, and hygiene (WASH) behaviours and catalysing community led solutions to change behaviours. Between June and August 2013, photovoice was conducted with eight (8) women in Usoma, a lakeshore community in Western Kenya with a follow-up community meeting (baraza) in May 2014 to discuss findings with the community members and government officials. In the first part of the study, photovoice one-on-one interviews were used to explore local perceptions and practices around water-health linkages and how the ecological and socio-political environment shapes these perceptions and practices. This paper, which is the second component of the study, uses photovoice group discussions to explore participants' experiences with and (re)action to the photographs and the photovoice project. The findings illustrate that photovoice was an effective CBPR methodology for understanding behaviours, creating awareness, facilitating collective action, and engaging with local government and local health officials at the water-health nexus.

## 1. Introduction

Over the past three decades, systematic reviews and meta-analysis of water, sanitation, and hygiene (WASH) interventions in poor communities with unsafe water and inadequate sanitation have shown that interventions can reduce the risk of diarrheal diseases [1–4] and other water related diseases such as ascariasis, dracunculiasis, hookworm infection, schistosomiasis, and trachoma [5, 6]. Thus, the provision and promotion of low cost WASH technologies—at the individual, household, and community levels—are regarded as a key strategy for reducing water-borne and water-related diseases in resource poor settings, particularly in low and middle income countries [7]. Examples of such interventions that have been found to have an impact on water-borne and water-related diseases include hand washing with soap [8, 9], integrated water supply and sanitation [10], household and village level treatment, water storage and hygiene education [11–13], and face washing [14–16]. In addition, it is well recognised that, for “hardware” interventions to have significant impacts on population health and contribute to global health, technologies need to be complemented by “software interventions” such as sustainable behaviour changes over time [7]. However, there are several reported difficulties in the implementation, adoption, sustenance, and scaling out of WASH behavioural interventions [17–20].

Key limitations identified to affect the design and implementation of behaviour change interventions include the lack of clear statements about—and attention to—the processes and strategies for implementation such as type of message given and medium used [17]; limited understanding of factors that influence WASH behavioral change adoption and sustenance [17, 21]; and limited attention to theories when undertaking behavioral change interventions [22]. In order to address some of these challenges, a number of researchers have suggested engagement with and use of multiple theories including social capital theory, communication theory, social learning theory, and other behavioural theories in the design and implementation of interventions [17, 22, 23]. Further, Briscoe and Aboud [17] in a review of behaviour change techniques targeting bed nets, hand washings, face washing, and complementary breastfeeding in developing countries have highlighted the need to use multiple techniques to engage participants. They classified these techniques into six categories that can be used to engage participants at the individual, social, cognitive, or sensory levels. These include information techniques, performance-based techniques, problem solving technique, social support techniques, and material techniques and using forms of media. From both research and theoretical perspectives, Briscoe and Aboud [17, page 619] further suggest the use of “social action research and theories that aim to change norms from the top-down or bottom-up of a community.” Thus, linking behavioral change techniques with community based participatory research (CBPR) may be necessary for enabling and sustaining behaviour change at the community level [17, 24]. In addition, the use of CBPR approaches can help address the gaps between theory, research, and practice in health behavioural change research [25]. At the heart of CBPR is the development of collaboration between researchers and communities that utilises shared knowledge and experience to catalyse social change [25].

This paper reports findings from an emerging CBPR method, photovoice, that uses “participant-employed photography and dialogue” to create social change [26, page 1393]. Specifically, the paper seeks to explore the use of photovoice as a behavioural intervention within WASH research projects. The study was conducted in Usoma, a lakeshore community in western Kenya. The paper is part of a broader Attitudes, Practices and Empowerment (KAPE) project implemented in the Lake Victoria Basin in East Africa by the United Nations University Institute for Water, Environment and Health (UNU INWEH), Canada, that aims to (a) educate and build capacity of local communities around water and health and (b) educate local communities on key elements of maintaining high levels of population and public health in the context of safe water. In order to meet these broad objectives, photovoice was previously used within the same community to map and understand community disparities relating to water, sanitation, and health [27] and to explore local perceptions and practices around water-health linkages and how the ecological and sociopolitical environment shapes these perceptions and practices [28].

### 1.1. Community Based Participatory Research, Photovoice, and Health Behavioural Interventions

Photovoice is regarded as a CBPR methodology that can be used to foster trust and capacity building for community led solutions to environment and health issues [26, 27]. Green et al. [29, page 419] defined CBPR as a “systematic inquiry, with the collaboration of those affected by the issue being studied, for purposes of education and taking action or effecting change.” CBPR, with its “family” of approaches such as participatory research, participatory action research, and collaborative inquiry, has its origin in action research [30] which seeks to facilitate change through theory testing and practical interventions and actions [31]. With regard to WASH behavioural interventions, CBPR expands the potential for bridging the worlds of research, policy, and practice. That is, CBPR can be used for research and development, implementation, and dissemination of effective health interventions in diverse communities and resource settings [32]. Further, through techniques that foster critical reflection, dialogue, and mutual learning, CBPR can be employed to address power imbalances and facilitate learning, action, and capacity building for marginalised populations [26].

As an emerging CBPR methodology, photovoice promotes social action by equipping communities to participate in the identifications and analysis of local problems. Through photography, participants are able to identify, represent, discuss, and find solutions to their everyday environment and health problems [26]. The use of photovoice in health and environment research is greatly influenced by the works of Wang and her colleagues who initially used the principles and techniques to enable Chinese village women to photograph challenges to their everyday health and well-being [33–36]. Wang [33] identified three main theoretical foundations of photovoice: Freirean-based education techniques [37], feminist theory and practice [36], and documentary photography [38]. Recognising that the lack of safe water and adequate sanitation places a disproportionate health and social burden on women and children [21, 28, 39], photovoice could be a powerful method for exploring a wide range of challenges around the water-health nexus. Though many studies have used photovoice to explore health perceptions, behaviours, practices, and interventions [40–42], literature documenting the use of photovoice in WASH research particularly in the context of sub-Saharan Africa remains limited, and an exception includes Levison et al. [27]. However, within the water and sanitation literature, photovoice can be regarded as an integral part of participatory research and intervention protocols that ensure that communities are involved in researching, planning, and implementation strategies to improve their health and well-being within the context of water. Some of these participatory protocols that have shown practical importance include public participatory geographic information systems [43–45] and community led total sanitation [46].

## 2. Research Context

### 2.1. Water and Sanitation in Usoma

**Figure 1:**
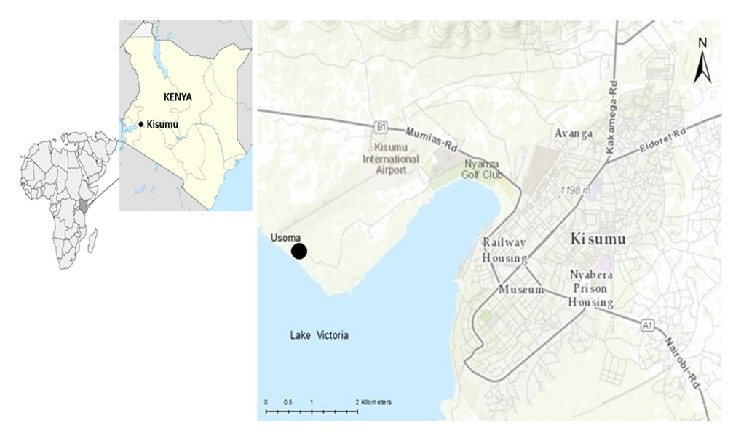
Study site: Usoma, Kenya [28].

Usoma is located about 15 km from Kisumu, the third largest city in Kenya. Though located on the shore of Lake Victoria (the second largest freshwater lake in the world), the people had no access to safe water at the time of this study. The nearest safe water source was a tap located 3 km away on the premises of a Coca-Cola bottling plant and was perceived to have high chlorine content and the few wells and boreholes that existed were mostly dried up or contaminated [28]. Thus majority (86%) of the community depends on the lake for their domestic water needs [39]. This has resulted in high incidence of water-borne and other water related diseases. For example, over 90% of school children are found to have schistosomiasis infections [47]. In addition to contamination by human and animal waste, the lake over the years has been contaminated through adverse industrial activities such as dumping of waste and sewerage. With regard to sanitation, a survey conducted indicated that about 42% of households in the community practice open defecation, with pit latrines being the most commonly reported sanitation facility in the community [39]. Over the years, marginalisation of the community (such as inadequate provision of social services by municipal authorities and government agencies) together with unemployment has served as major barriers to improving water and sanitation conditions [27, 28, 48, 49].  Figure 1 shows the locations of Usoma.

## 3. Methods

This section illustrates how photovoice was used as a CBPR method within a larger research program. In June 2013, a community baraza (community meeting) was held to discuss the broad objectives of the research and seek approval from community leaders. Prior to this, there has been a long standing relationship and research collaborations between Kenya Medical Research Institute (KEMRI), UNU-INWEH, and the Usoma community. From the baraza, a village elder was elected to work with the research team in order to facilitate access to and recruitment of participants. Women were recruited in line with the theoretical foundations of photovoice [36] as well as consideration for the gender related impacts of access to safe water and adequate sanitation. That is, women typically bear the greatest burden of providing water for households in most parts of sub-Saharan Africa, have less decision-making authority, and are equally at risk from both health and social impacts associated with water collection and lack of access [21, 28]. Participants were recruited using snowball sampling techniques [50]. Two women were first identified based on previous local contacts and their involvement in community activities. These women were then asked to contact other participants who had lived in the community over 1 year and would be interested in participating in the study. Eight women (including two community health volunteers) with ages ranging between 28 and 55 years were recruited for the study between June and August, 2013. The sample size was adequate to generate rich data (photographs and narratives) and thick description of the issues studied, while at the same time maintaining a manageable number of photographs for the group discussion [28].  Table 1 shows a summary of the sociodemographic profile of participants.

Once participants had agreed to participate in the study, a group training session on ethical considerations in the research process and taking of pictures as well as technical use of disposable cameras was provided to all the participants. Participants were then asked to take pictures of what for them best represented* attitudes and practices around water and sanitation that influence health in the community*. Photos were taken over 8 days after which the cameras were collected and the photographs developed. In total, participants took between 16 and 26 photographs. One set of photographs was given to each participant as a token of appreciation for their contributions. Each participant then chose four photographs that best represented her views to be used for a group discussion. Thus 32 photos were used for the group discussion (that lasted 7 hours) involving all the eight women. During the discussions, all the photos were spread on a large table and participants sat around the table to broadly discuss their experiences and reactions to the photographs and the photovoice project in general. The group discussion was facilitated by the lead author with translation into DhoLuo (the local language widely spoken in the community) provided by the third author. Before the discussion, the same photographs were initially used as a basis for one-on-one interviews to capture sociopolitical drivers of water-related practices, findings from which are reported in Bisung et al. [28].

The group discussion was audio-recorded, transcribed verbatim, and analysed with the aid of NVIVO 10, a qualitative analysis software package (detailed description of the data analysis procedure and sample photographs used in the discussions are provided as supplementary data in Bisung et al. [28]). After the analysis, a baraza was held in May, 2014, to share the preliminary results and elicit feedback in order to enhance rigour. The baraza further gave the community an opportunity to discuss ways of finding sustainable community led solutions. Present at the baraza were provincial leaders, public health officials, researchers, school children, and community leaders. The public health officials delivered health messages and the school children performed poetry recitals on open defecation and hygiene practices. Bars of soap were also presented to all community members present as a motivation and reminder for people to attempt or adopt hygiene practices as well as a token of appreciation from the researchers. The community baraza was facilitated by the third author and conducted in both English and DhoLuo. The study received ethical clearance from the University of Waterloo Ethics Review Board and the Ethics Review Committee of KEMRI (SSC Protocol # 2468).

## 4. Results

From the group discussion, three major themes emerged: awareness, immediate (re)actions, and planned actions. These themes illustrate how women, through their photographs and narratives, can generate community led WASH interventions.

### 4.1. Awareness

Participants reported that the photos served as prompts to certain behaviours and practices in the community. Though such practices existed for long, some participants were not aware of them. They discussed how the photos made them realise the influence of some everyday practices on their health. For example, this woman describes how the photographs and discussions made her aware of other behaviours beyond what she is familiar with:

*I am happy with this photos and discussions because I am now aware of some few other behaviours happening in the village which I was never aware of. I saw children drinking water right from the lake where their colleagues were swimming. (Participant #8)*

Similarly, others mentioned they did not realise how dangerous (dilapidated) some pit latrines were and especially how such latrines were located close to water collection points:

*I never knew about the existence of such toilets. That bad toilet next to an open well where people fetch water. I have never known or imagined that such a thing existed in this village. (Participant #6)*

*I was disturbed by what is happening around that water point, where the surrounding was not clean and people were still lining to get water from the same source. Also close to that water point are open places with stagnant water where children were swimming. That is a very bad practice which I never took notice of. (Participant #1)*

Further, other participants knew about the existence of certain behaviors but did not realise such behaviours were widespread in the community. For example, participant #6 said she learned a lot because she knew people practice open defecation but never realised it was so widespread until she wanted to photograph a pit latrine and could not find one within a reasonable distance:

*I realised most people practice open defecation because there are not enough toilets in the village. I wanted to take a picture of a pit latrine but had to walk for a long distance without getting any. It means most houses I bypassed on the way practice open defecation. A lot still needs to be done in terms of putting up more latrines to stop open defecation and I am happy this came up a lot in the pictures. (Participant #6)*

Similarly another participant realised most households did not treat their water before using:

*Despite the sanitation and health education we give to community members, I realised most homes still do not treat water from the lake. I was disturbed about that! (Participant #2)*

Though some participants were surprised about the scale of these behaviours and practices, others maintained most of them were common in the community and emphasised the need for the collective effort of all community members, as this woman mentioned:

*The community in general is not environmentally safe, and most parents also do not really look after or teach their children good practices. Children are allowed to do whatever they want inside the lake from swimming to bathing to drinking all in the same place. This doesn't pertain to only specific children or households, most children do that. We need to collectively address that. (Participant #3)*

Other participants stated they saw and learned more about their common practices than their differences, for example:

*I learned more about the practices we have in common, everywhere I went was virtually the same story. Either people are using unsafe water without treating or practicing open defecation. There are some few exemption but those are few. We really have common problems and need to find common solution. (Participant #4) *

The above observation by participant #4 reinforces the widespread nature of water challenges in the community and how photovoice can reveal commonalities among community members. Making people aware and conscious of these commonalities can be an important step towards facilitating collective actions.

### 4.2. Spontaneous and Immediate (Re)Actions

The discussions further explored participants' actions and reactions to some of the practices they encountered during the photo taking. Though participants were not instructed to take any action or react to practices and behaviours during the process, some reported taking spontaneous decisions to educate people and stop children from certain negative practices and having meaningful discussions on how to find solutions to common negative behaviours and practices. As described below, this woman said she used the photo taking as an opportunity to discuss water treatment practices:

*When I went to take pictures of women walking to the lake, we also discussed how to treat water at home. I think the women all agreed that so many people have stomach problems because of the unsafe water most of us are using. So it was an opportunity to discuss our common problems. (Participant #2)*

Aside from these spontaneous water treatment discussions, some participants said photo taking gave them an opportunity to gauge the level of interest and knowledge of community members on open defecation. Even though it is usually difficult talking about open defecation with people outside one's extended family, some participants talked freely about it even with “strangers” during the photo taking:

*It is sometimes difficult talking about open defecation to outsiders but I was able to discuss defecation practices freely. The people did realise it is bad to defecate in the bush because flies can transmit germs back into the house. (Participant #7) *

Further, most participants reported advising children or stopping them from certain practices during the one-week photo taking period. While some participants reported taking these actions (advising children) on a daily basis, others said the photovoice made them more conscious about children's behaviours and negative practices:

*I saw children standing on top of a well and drawing water which I realised was risky and dirty. I advised them not to stand on top but on the ground, and also told them the water should also be treated when they get home. (Participant #2)*

Similarly, another woman used the opportunity to teach children how to sieve water:

*Sometimes children are left alone to treat or sieve water and they mostly do it wrongly. I advised those children you can see in this picture [picture of two girls sieving water] the proper way to sieve water. The photovoice gave me that opportunity otherwise I would not have even gone to their house. (Participant #8)*

### 4.3. Planned Actions

The photovoice process (including training, photo taking, and group discussion) gave participants an opportunity to discuss and plan future interventions. Though proposed actions varied in nature, most participants emphasised the need to involve village leaders and the whole community. The kind of planned actions explored at the photovoice discussions exemplified the importance of CBPR methods in capacity building and social action. For example, in response to a question about why children stand inside the lake to do laundry, a practice that exposes many children and women to schistosomiasis infection, this woman said:

*Maybe most people haven't thought about it and just feel it is easy to just wash inside the lake… it is something we can tell the village elder and chief to organise a baraza so that we discuss ways of stopping it. (Participant #2)*

Another participant also added:

*I never noticed these dangers until I saw the picture, at the next Baraza we can discuss it further with the whole community or we can even start at our women's group level before the next community baraza. (Participant #1)*

Similarly, another participant gave the following suggestion regarding the continuous use of a dilapidated pit latrine that had not been cleaned for a while:

*If people using it can come together and decide to look after it, it will be good. After this meeting, we will tell the village elder to call a baraza and we will decide what to do. Closing it may be better since it is almost full. I am part of those who use it. I think that is a very bad practice. (Participant #8)*

From the above quotations, it is clear that some participants were implicated in some of these practices and thus felt some kind of obligation to act. For example, this was a participant's response to a boy standing on top of an open well and drawing water:

*I also use this well, I will tell my neighbours to contribute so that we buy something to always cover it. We can also stop people drawing water from standing on top of the well, but rather on the ground in order not to pollute the water. (Participant #5)*

Though the research team have not done a follow-up study to evaluate the level of implementation or success of these planned actions and reactions, the community feedback and dissemination signaled a strong sense of enthusiasm and desire among community members to address water and sanitation challenges. For example, community leaders indicated that some initiatives have been taken following our research. These included better cooperation among various groups in the community, completion of a water and sanitation block, and increased participation in the activities of the Usoma Water and Sanitation (UWASH) Committee.

## 5. Discussion

### 5.1. Understanding WASH Behaviours

Photovoice provided an opportunity for researchers to fully understand the complexities of water related behaviours in the community that other research methods such as surveys and interviews may not fully capture. Photovoice was able to capture both the social dimensions of behaviours such as open defecation and the impacts they have on the community, particularly on women and children. Further, the photographs generated critical face-to-face dialogue around these behaviours partly because both the researcher and participants were able to visualise and reflect on the behaviours. For certain behaviours that participants found difficult explaining or discussing with researchers, the photographs acted as a medium of communication between the researcher and participants and served as prompts for participants to easily remember issues they wanted to talk about [51]. Thus, photovoice can produce useful information about behaviours that participants may otherwise forget. Further, photo discussions enable participants to critically analyse photos thus revealing useful dimensions to specific issues. For example, a photo may not contain new information about WASH behaviours to a particular participant or the actual photographer but can trigger meaning and conversations during group discussion that highlights other issues in the photo that may not be apparent even to the photographer. For example, when a participant showed a picture of a child sieving water to highlight water treatment practices and knowledge among children, the photo rather triggered discussion around the disadvantages of children sieving water without any supervision from adults.

### 5.2. Effecting Behaviour Change and Practices

The findings from this paper suggest that photovoice can be used to facilitate behaviour change through creating awareness and triggering immediate (re)action and planned actions. Photovoice was able to generate instant behavioural messages through participants' own photos that were context relevant and participant driven. In this regard, participants become conscious of the health impacts of their everyday practices and started to think about ways of addressing them. Thus photovoice was able to overcome problems associated with how to deliver simple, understandable, and memorable messages to participants during interventions. While this study was conducted with only eight (8) women, the process has the potential to inspire participants to become advocates within their households and the community in general thus facilitating diffusion of the issues discussed.

Though a number of change theories and models such as theory of reasoned action [52], the health-belief model [53], and the stages of change model [54, 55] have been used in behavioural change interventions, researchers have pointed to the need to consider other theories such as social learning theory, diffusion of innovation theory, social capital theory, and collective action theory that provide opportunities to address changes at different levels: individual, household, and community levels [21, 22, 56–58]. As noted by Figueroa et al. [57], the prevention of a disease or achieving maximum benefits from a behavioral intervention may only be successful through strategies that foster collective actions beyond the individual or household level. For example, evidence has shown that risks of diarrhoea attributed to WASH can be reduced through improvements in excreta disposal, hygiene practices (hand washing with soap), and water quality. However because diarrhea is caused by various pathogens, transmitted by various routes and associated with various potential confounding factors [59], behavioral interventions can be most effective if responses are both individual and collective. Thus both individual change techniques and strategies as well as collective change techniques and strategies appear important for WASH related behaviours and diseases. From this study, photovoice was effective at delivering individual messages as well as facilitating future collective actions. Photovoice thus appears to be an effective method for eliciting both individual and collective behaviour responses. However, we suggest further exploration in different intervention settings in order to identify best intervention strategies and temporal outcomes.

Further, photovoice can utilise several behaviour change techniques (modes of engagement) to engage participants when integrated with other community based activities. From the matrix of change techniques identified by Briscoe and Aboud [17], this study employed a number of them in order to achieve active participant engagement and address different aspects of the behaviours. The following techniques that were included are worth noting:* problem solving *(e.g., identifying barriers and facilitators to open defecation during interviews and baraza),* media* (e.g., using photos to provide information as well as a means for critical consciousness),* materials* (providing soap to community members during feedback baraza to encourage people to attempt hand washing), and* information giving* (e.g., using the baraza as an opportunity for public health officials and leaders to deliver WASH messages). The use of these multiple techniques is capable of engaging community members at the social level (social support and media), sensory level (material and media), and cognitive level (information giving), thus consolidating learning and practicing of positive behaviours [17].

### 5.3. Challenges of Photovoice in CBPR Research

Photovoice in CBPR research and especially research that seeks to generate social action has several challenges partly because of issues associated with what can and cannot be photographed [26, 39]. For example, while there was considerable discussion around open defecation, ethical considerations limited the kind of photos participants could take. To partly address this challenge however, group discussions were used to explore participants' perspectives beyond what was visibly captured in the photos. Further, as was done in this study, photovoice was complemented with other “traditional” research methods such as surveys in the broader research project to fully explore all behaviours and practices related to collective action in the community [39]. In addition to the above challenges, researchers and participants in photovoice projects sometimes have to deal with the challenge of selecting a specific number of “best” photos for face-to-face interviews or group discussion due to the large number of photographs taken by participants most times. A major limitation associated with this selection process is that photovoice sometimes produces so many pictures that researchers may find compelling or relevant to the study or intervention objectives but cannot use them all because they are not selected by participants and thus do not have accompanying narratives [43]. This can create a scenario where the “unselected” is denied attention when designing and implementing larger interventions. Another recurrent challenge in our project relates to participants desire to take photos of other important things/events (e.g., pets, children playing, new bicycle, etc.) in their lives that were however not related to the research project. Though we did not record more than 2 of such photos per participant, other inappropriate uses of disposable cameras to take sensitive pictures of participants' family members have been reported by Clark-Ibáñez [51].

Finally, researchers also require reasonable time to build trust and collaborative partnerships with local research institutions. In the case of this study, the research team and local partners have been working in this community for over a decade studying various environment and health issues and building mutual trust. Further, both researchers and participants spend time in training, photo taking, interviewing, group discussion, and community meetings to effect change. A great deal of commitment may therefore be required from both participants and researchers in order to successfully implement a photovoice project.

### 5.4. Implications for Health Behaviour Change

In summary, we emphasise three main implications of our findings for health behaviour change. First, community participation can help in the implementation of culturally acceptable and compelling health interventions [60]. Photovoice can be a useful tool for behaviour change interventions in this regard as it focuses on community led identification of problems, embedded within preexisting social structures, and amplifies the voices of those most affected. These voices—which often remain silent—can catalyse collective action to change behaviour and related community practices. Second, behaviour change projects should incorporate an evaluation mechanism in order to make explicit the links between interventions and health impacts. Though this aspect was missing in our study, making these links explicit will help to identify important determinants of behaviour change in order to design appropriate strategies that can generate maximum public health impacts in different contexts. As noted by Panter-Brick et al. [60, page 2824] clearly distinguishing and linking “intention to change, actual behaviour change, and subsequent health impact” provides useful information for designing, implementing, and evaluating interventions for cultural and social effectiveness. Finally, despite the existence of negative WASH related behaviours, there was a demonstration of adequate knowledge of the health impacts of these behaviours as well as a strong desire to address them both at the individual and community levels. However, this desire has not been translated into concrete actions with identifiable outcomes over the years due to structural barriers. Reported barriers to behaviour change in the community include general lack of water to enhance adoption and diffusion of hygiene behaviours and lack of financial resources to invest in water and sanitation facilities [28]. Thus, individual and community intent to change behaviours as demonstrated in the group discussions reported here may not be sufficient to result in sustainable behaviour changes in the face of these barriers. Given the limited financial resources in the community and other poor settings, sustainable behaviour change may require concrete external support and investment to provide the needed facilities (household latrines, safe drinking water, treatment products, etc.) that support adoption and diffusion [56, 61].

## 6. Conclusion

This study contributes to our understanding of photovoice as a method for understanding WASH behaviours and effecting behaviour change. Photovoice ability to foster community oriented solutions rather than individual solutions in the long run is important for diffusion and adherence to positive practices. In this study, photovoice provided an opportunity for community members to discuss their own behaviours and for researchers to appreciate participant's knowledge around water-health linkages. Giving power and control for the community to identify and discuss how to tackle these challenges enhanced trust between the researchers and the community [26] as well as a sense of ownership over the research process, outcomes, and collective actions.

According to Aboud and Singla [22], reviews of behavior change interventions, especially those related to the Millennium Development Goals (MDGs), provide evidence of effective solutions/techniques and failures as well. To address these failures, some researchers have drawn attention to the need to engage in theory driven research and the use of multiple theories and techniques to inform interventions in order to effect change [22, 23]. Thus photovoice could be very useful for behavioural research and interventions considering its strong theoretical foundations (Freirean-based education, feminist theory, and documentary photography) and cultural appropriateness to vulnerable groups [26]. In addition to its strong theoretical foundation, photovoice incorporates several techniques (photos, community meetings, interviews, discussions, and engagement with health officials and locals) that address cognitive and social aspects of behavioural interventions.

In terms of future direction, a follow-up photovoice study could be designed to evaluate changes in behaviours. In this regard, photovoice can be used for monitoring and evaluating WASH interventions [43]. For example, with financial support from UNU-INWEH and Rotary Club of Hamilton, Canada, as well as private donations, the community has been able to construct a sanitation facility and has facilitated the extension of municipal water to the facility for vending to community members [49]. Though these facilities were not constructed at the time of this study, future studies could evaluate their impacts on WASH behaviours. Further, we suggest that other WASH researchers interested in CBPR approaches should consider using photovoice in other cultural and ecological contexts to expand the nascent literature as well as test the effectiveness of photovoice in effecting WASH behaviour changes. In WASH and development practice, incorporating photovoice into the design, implementation, monitoring, and evaluation protocols of projects and programs can go a long way to provide answers to many unanswered questions.

## Figures and Tables

**Table 1:** Summary description of participants.

Participant's #	Age (years)	Education	Occupation	Length of stay in the community (years)
Participant 1	28	High school	Unemployed	5
Participant 2	33	Standard eight	Fish seller	12
Participant 3	22	Standard eight	Housewife	7
Participant 4	49	Standard seven	Seamstress	23
Participant 5	54	High school	Fish seller and a community health volunteer	30
Participant 6	34	Standard eight	Unemployed	6
Participant 7	43	Standard eight	Businesswoman	12
Participant 8	39	None	Housewife	24

Note: participant # is used to ensure anonymity of participants.
